# Deep learning classification of drainage crossings based on high-resolution DEM-derived geomorphological information

**DOI:** 10.3389/frai.2025.1561281

**Published:** 2025-05-13

**Authors:** Michael Edidem, Bill Xu, Ruopu Li, Di Wu, Banafsheh Rekabdar, Guangxing Wang

**Affiliations:** ^1^School of Earth Systems and Sustainability, Southern Illinois University, Carbondale, IL, United States; ^2^Carbondale Community High School, Carbondale, IL, United States; ^3^Department of Computer Science, Portland State University, Portland, OR, United States

**Keywords:** GeoAI, drainage crossing, CNN, XAI, hydrography

## Abstract

High-resolution digital elevation models (HRDEMs) from LiDAR and InSAR technologies have significantly improved the accuracies of mapping hydrographic features such as river boundaries, streamlines, and waterbodies over large areas. However, drainage crossings that facilitate the passage of drainage flows beneath roads are not often represented in HRDEMs, resulting in erratic or distorted hydrographic features. At present, drainage crossing datasets are largely missing or available with variable quality. While previous studies have investigated basic convolutional neural network (CNN) models for drainage crossing characterization, it remains unclear if advanced deep learning models will improve the accuracy of drainage crossing classification. Although HRDEM-derived geomorphological features have been identified to enhance feature extraction in other hydrography applications, the contributions of these features to drainage crossing image classification have yet to be sufficiently investigated. This study develops advanced CNN models, EfficientNetV2, using four co-registered 1-meter resolution geomorphological data layers derived from HRDEMs for drainage crossing classification. These layers include positive openness (POS), geometric curvature, and two topographic position index (TPI) layers utilizing 3 × 3 and 21 × 21 cell windows. The findings reveal that the advanced CNN models with HRDEM, TPI (21 × 21), and a combination of HRDEM, POS, and TPI (21 × 21) improve classification accuracy in comparison to the baseline model by 3.39, 4.27, and 4.93%, respectively. The study culminates in explainable artificial intelligence (XAI) for evaluating those most critical image segments responsible for characterizing drainage crossings.

## 1 Introduction

Detailed surface hydrological features are essential for environmental management, including habitat and ecological conservation, as well as flood mitigation and water quality preservation (Schultz et al., 2017; Good et al., 2015; Pringle, 2001; Dadson et al., 2017; Simley and Carswell, 2009). In recent decades, these features are often obtained from High-Resolution Digital Elevation Model (HRDEM) datasets, primarily being generated using Light Detection and Ranging (LiDAR) and interferometric synthetic aperture radar (InSAR) technologies (Sharma et al., 2016). The use of these HRDEMs has been found to improve the efficiency, accuracy, and consistency of hydrological features (Xu et al., 2021; Stanislawski et al., 2021; Edidem et al., 2025).

However, the extraction of hydrological features from HRDEMs faces significant challenges due to anthropogenic barriers, such as roads and bridges, often referred to as “digital dams” (Duke et al., 2006; Li et al., 2013; Sofia et al., 2014; Wu et al., 2024). For example, at drainage crossing locations, HRDEMs typically represent the elevation of the top surfaces of these barriers but miss the underlying culverts through which water flows (Poppenga and Worstell, 2016). This limitation presents a significant challenge for fine-scale hydrologic modeling in smaller watersheds. Digitally represented flow barriers without properly modeled drainage crossings can obstruct downstream flow, leading to the formation of extensive upslope artifact depressions (Van Nieuwenhuizen et al., 2021). The absence of accurately mapped drainage crossings such as culverts and bridges may cause simulated drainage flowlines to terminate prematurely or cross roads at incorrect locations (Poppenga and Worstell, 2016). As a result, simulated streams often show drainage crossing misalignment and disrupted hydrological connectivity (Li et al., 2013; Barber and Shortridge, 2005).

To ensure continuous surface flowline modeling and connectivity, HRDEM must be effectively free of depressions, resulting in their preprocessed “hydrologic” version. This preprocessing step often begins with enforcing continuous downslope drainage patterns by removing topographic sinks (Lidberg et al., 2017; Lindsay, 2016; Reuter et al., 2009). Common techniques include depression filling methods (Wang and Liu, 2006; Wang et al., 2019), which involve filling depressions caused by embankments, ditches, or slopes, using various algorithms that yield comparable results. Depression filling can alter the original terrain, especially in low-lying areas with dense road networks and drainage crossings, resulting in erroneous flowlines (Schwanghart et al., 2013). An alternative approach is the breaching method, which involves cutting through barriers like roads or bridges to enforce flowlines (Sofia et al., 2014), and stream burning (also known as hydrological enforcement), which uses drainage crossing linear segments to cut roads open to enhance hydrologic connectivity (Li et al., 2013; Lindsay and Dhun, 2015). However, both breaching and burning methods are ineffective at accurately removing flow barriers without precise drainage crossing locations (Lessard et al., 2023). These limitations are particularly problematic for HRDEMs where fine-scale features are critical for accurate hydrological modeling.

Wu et al. (2024) demonstrated that effectively identifying and processing flow barriers within HRDEMs is critical for enhancing hydrography mapping accuracy at fine scales. Several previous studies have demonstrated that including drainage crossing locations can successfully breach flow barriers and improve the spatial precision of HRDEM-derived watershed boundaries and flowlines (Li et al., 2013; Aristizabal et al., 2018; Bhadra et al., 2021; Lindsay and Dhun, 2015). Despite this, datasets representing drainage crossing locations are often unavailable or of poor quality, making it challenging to obtain accurate and fully connected hydrological features. Current methods for obtaining culvert data such as manual on-screen digitization (Shore et al., 2013) or field surveys (Lessard et al., 2023; Hao et al., 2011; Arsenault et al., 2023; Açıl et al., 2023), are costly and labor-intensive.

Recent advancements in software and hardware capabilities, along with the development of sophisticated deep learning (DL) algorithms, have accelerated the rapid expansion of GeoAI, a field that leverages artificial intelligence for geographic data analysis. The increasing availability of high-resolution geospatial data combined with the emergence of scalable computation platforms, continues to drive this advancement. DL has emerged as a powerful tool for predictive analytics and pattern recognition, particularly within environmental applications. For example, Xu et al. (2018) utilized a 3D CNN-based data fusion model to integrate 3D features from LiDAR and multitemporal images, achieving high-precision classification and extraction. Stanislawski et al. (2018) trained a CNN to extract comprehensive road and stream valley features from HRDEMs using a combination of existing road and stream valley data. Li et al. (2020) employed a DL strategy to classify complex and transitional landforms in the Loess Plateau using integrated data sources, including DEMs, imagery, and terrain derivatives such as slope and aspect. Iqbal et al. (2022) developed CNN algorithms specifically for identifying debris-related blockages in drainage culverts through the classification of HRDEMs. These examples demonstrate the versatility of DL in addressing complex geospatial challenges by leveraging diverse data sources.

More recently, advanced DL models have shown remarkable improvements in performance for specific geospatial tasks. Yin et al. (2022), for example, proposed the EfficientNetv2 model, for land-use classification. The results demonstrated improved performance compared to VGG, MobileNetV2, ResNet34, and EfficientNet-b0. Similarly, Jeba and Chitra (2023) utilized the EfficientNetv2 and Self-AttentionNetv2 models, resulting in improved performance compared to ResNet-50 and CNN for the SEN12-FLOOD dataset. This dataset includes satellite images from both Sentinel 1 and Sentinel 2 satellites specifically for detecting floods. These advanced architectures demonstrated significant improvements in accuracy and reliability compared to earlier models like ResNet-50 and standard CNNs. Such advancements highlight the growing potential of DL to address increasingly complex and specialized geospatial problems, while leveraging its general strengths in accuracy, reliability, and consistent workflows across time and space (Xu et al., 2018).

In hydrography, the integration of CNNs combined with HRDEMs, and their geomorphological derivatives have demonstrated significant potential for detecting topo-hydric features. Geomorphic layers such as topographic position index (TPI), slope, curvature, and openness are frequently utilized to provide critical input data for DL models, enabling the classification of terrain types, hydrological zones, and other geospatial features. For instance, Stanislawski et al. (2018) demonstrated the utility of HRDEMs in identifying road and stream valley features, while Xu et al. (2021) utilized geomorphological derivatives like topological curvature, TPI and positive openness for the detection of fine-scale hydrologic streamlines. In another studies, Barlow et al. (2022) found that slope and elevation were among the most influential features for mapping stream boundaries, highlighting their potential for classification models. These examples showcase how geomorphological layers derived from HRDEMs provide robust input data, underscoring their value in advancing classification tasks within geospatial analysis.

DL algorithms, particularly CNNs, have shown substantial promise in detecting drainage crossings, such as culverts, within HRDEMs. Talafha et al. (2021) demonstrated this capability within a specific watershed area, suggesting the approach could have broader geographic applicability. Building on this, Wu et al. (2023) investigated optimal data inputs for drainage crossing classification, testing a combination of HRDEM, Normalized Difference Vegetation Index (NDVI), Normalized Difference Water Index (NDWI), and National Agriculture Imagery Program (NAIP) aerial orthophotos. Their results showed that CNN models trained solely on HRDEM data achieved a notable accuracy of 93.33% and demonstrated excellent transferability across diverse geographical contexts. Edidem et al. (2025) further advanced this area by applying object detection models to HRDEMs in agricultural areas, demonstrating high accuracy and robustness across diverse watersheds. However, this study did not incorporate any HRDEM geomorphological derivatives. Despite these advancements, the potential impact of additional topographic features and the use of alternative or more complex DL algorithms remains an open question. This gap suggests further exploration could improve drainage crossing classification accuracy, especially by considering topographic intricacies and employing advanced DL techniques beyond traditional CNNs.

Although CNNs are prominent as a deep learning approach due to their exceptional ability to extract meaningful patterns from complex data, their intricate structures often render them as “black boxes,” limiting the understanding and transparency in their decision-making processes and raising concerns about the scientific reliability of their predictions (Kedron et al., 2021; Li and Arundel, 2022). This opacity in GeoAI research presents a barrier to reproducibility and understanding (Goodchild and Li, 2021). Explainable Artificial Intelligence (XAI) addresses these issues by revealing the factors influencing model, fostering trust and comprehension of AI systems. For example, Li (2022) compared XGBoost’s ability to capture spatial effects with traditional statistical approaches like the spatial lag model and multi-scale geographically weighted regression, employing SHapley Additive exPlanations (SHAP) for interpretability. The study found that XGBoost’s results were on par with those of traditional spatial models, suggesting that advanced machine learning methods can provide spatial insights comparable to conventional approaches.

Two primary XAI approaches have been developed for deep learning: global and per-decision explainable AI algorithms (Phillips et al., 2020). Global methods such as Testing with Concept Activation Vectors (TCAV) (Kim et al., 2017), approximate model behavior to identify influential concepts. Per-decision methods focus on explaining individual predictions, identifying patterns and concepts often using saliency maps, which visualize the importance of different regions in the input image influencing the final decision. For hydrography applications, XAI has significant potential to clarify how specific topographic features influence drainage crossing identification, making model predictions more interpretable for experts. By enhancing transparency, these methods can also improve confidence in AI-driven geospatial insights, supporting better-informed decision-making in environmental and geographical analysis.

This study aims to improve the accuracy of drainage crossing classification by evaluating the effectiveness and explainability of advanced DL models. The selection of input geomorphological features is based on their recognized utility for landform classification and feature extraction, as highlighted in various studies (Xu et al., 2021; Stanislawski et al., 2021). XAI method is integrated to ensure that the model predictions correspond to human-understandable concepts, improving both interpretability and trustworthiness. The results contribute to the best practices of hydrographic feature extraction, thereby increasing the precision and reliability of models for next-generation hydrographic mapping.

## 2 Materials and methods

### 2.1 Study area

**Figure 1:**
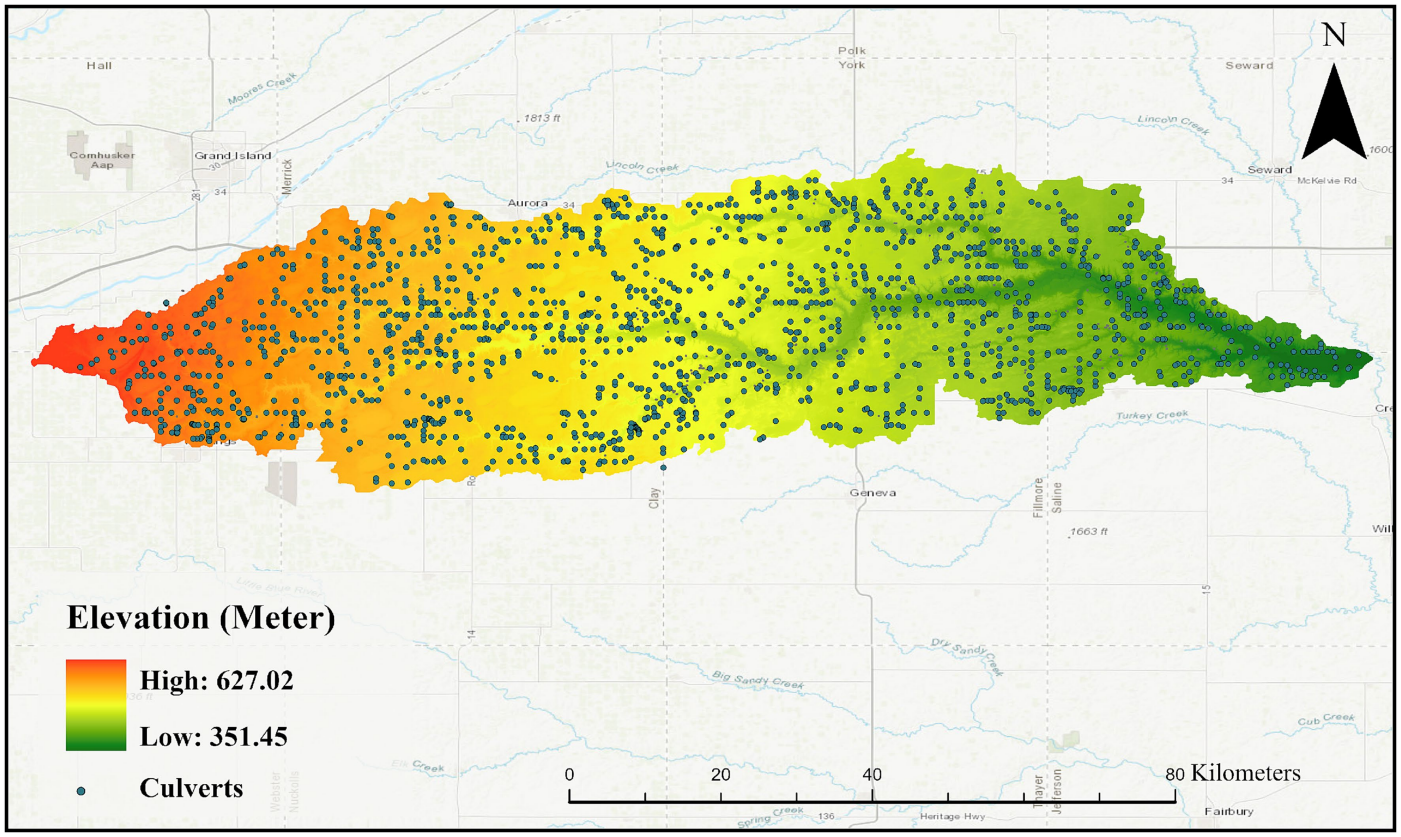
Topography of West Fork Blue Watershed in Nebraska with Culverts distribution.

This research focuses on the West Fork Big Blue Watershed, which is located in Nebraska and is depicted in Figure 1. Spanning approximately 3,471 km^2^, the region consists of a gently undulating loess plain with elevations ranging from 627 meters to 351 meters and is primarily utilized for row crop agriculture. The West Fork Big Blue watershed, predominantly situated in Seward County, Nebraska, is characterized by rolling hills and extensive row crops, with the Big Blue River flowing southeast. The diverse terrain of the watershed includes flat lands and moderate slopes, with elevations ranging from 352 meters to 627 meters, and it is marked by numerous depressional wetlands due to an underdeveloped drainage system (Stutheit et al., 2004). The watershed is also marked by a dense road network, which disrupts natural water flow and emphasizes the need for effective hydrologic feature management, particularly at culverts and bridges in HRDEMs. Additionally, the complexity of the terrain underscores the necessity of strategically breaching drainage barriers to maintain hydrological connectivity.

### 2.2 Datasets

This study leveraged the distinctive topographic patterns of elevated roads intersecting low-lying drainage channels (Gelder, 2015; Poppenga et al., 2010). These patterns serve as a crucial guide for manual digitization of flow barriers. Drainage crossings were designated as areas where natural streams or artificial canals intersect roads through underpass hydraulic structures such as bridges or culverts. By overlaying high-resolution aerial orthophotos, we identified and manually digitized drainage crossing locations, as shown in Figure 1. The model’s inputs comprise of individual image patches sampled from five different feature maps, each with a resolution of 1 meter. These feature maps are normalized versions of the five raster data layers, where each floating-point dataset within the study area is converted to a corresponding unsigned integer value scaled between 0 and 255 using Equation 1. Normalization ensures consistency across features, eliminating range disparities ranges (as shown in Table 1) while preserving relative differences and information integrity. For each identified drainage crossing, a 100-meter square bounding box was used to clip five-band samples of identical dimensions (100 m x 100 m pixels) as True samples. The dataset contains 4,044 image samples with an equal distribution of true and false samples. The five co-registered 1- meter resolution raster data layers are utilized for training, validation, and testing. This layer selection stems from a comprehensive literature review comparing elevation derivatives and nationwide-coverage optical imagery across various landscapes and utilization of such topographic layers in extracting hydrologic and geomorphic features (Xu et al., 2021; Stanislawski et al., 2021; Li et al., 2020).


(1) $$\text{Xnorm}=\frac{X-X_{\min}}{X_{\max}-X_{\min}}*255$$

**Table 1:** Summary statistics of raster images for the West Fork Blue Watershed, Nebraska.

Raster image name	Minimum	Maximum	Mean	Standard Deviation	Range
Digital elevation model (meters)	351.45	627.02	521.08	43.18	275.57
Geometric curvature	−24.26	53.96	−1.47	0.57	78.21
Topographic position index (3 × 3 window)	−131.24	16.43	−9.29	0.02	147.67
Topographic position index (21 × 21 window)	−147.22	15.98	−4.11	0.12	163.19
Openness (R10, D32) degrees	1.77	155.80	89.72	2.11	154.03

The raster layers include: an HRDEM and four layers are derived from the HRDEM to capture specific topographic features relevant to the classification task. Two Topographic Position Index (TPI) layers, calculated using window sizes of 21×21 and 3×3 cells, identify ridges, valleys, and flat areas (Deumlich et al., 2010). TPI emphasizes local elevation extremes in a DEM relative to nearby topographic features, highlighting ridges and valleys (De Reu et al., 2013). Zenith Angle Positive Openness (POS) with a 10-meter radius emphasizes drainage patterns and small streams (Doneus, 2013). Finally, Geometric curvature measures the curvature of a surface by combining curvatures in both the x and y directions and provides a detailed characterization of the surface’s shape by integrating the curvature values from multiple directions (Pirotti and Tarolli, 2010; Cazorzi et al., 2013). For instance, the mean curvature is often used and is calculated as the average of the maximum and minimum curvature at specific cells, or even as the mean value over all cells in a grid (Wood, 1996). In the context of DEMs, geometric curvature can be determined using various software tools. GeoNet, for example, applies a non-linear diffusion Perona-Malik filter to DEMs to reduce noise and enhance channel localization, subsequently calculating geometric curvature for the filtered DEM (Passalacqua et al., 2012; Sangireddy et al., 2016). The statistics of each raster layer are presented in Table 1.

Additionally, to assess the model’s spatial generalizability and sensitivity to positional bias, a subset of the true sample dataset, hereafter referred to as the off-center dataset was constructed by randomly shifting the image clipping polygons. This curated subset comprises 50 validated samples in which the drainage crossings are deliberately positioned away from the image center. This adjustment was necessary because, in the original training dataset, drainage crossings were consistently centered within sample images. By varying the spatial locations of drainage crossings, the off-center dataset allows a more rigorous evaluation of the model’s adaptability to different target positions and its capability to generalize beyond its training conditions.

### 2.3 Model development

**Figure 2:**
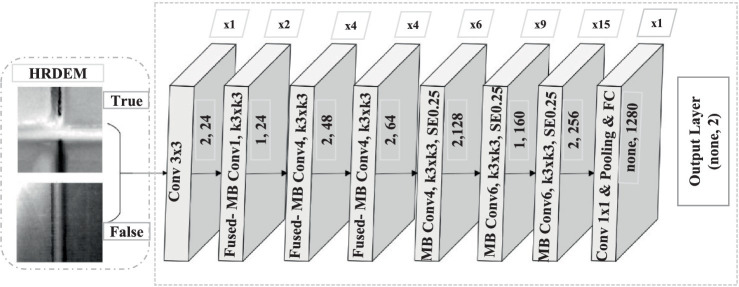
Architecture of EfficientNetV2 for classifying drainage crossings.

EfficientNetV2, an advanced CNN architecture, was chosen for its ability to achieve both high accuracy and efficiency training. EfficientNetV2 utilizes a series of convolutional and pooling layers to optimize depth, width, and resolution for optimal performance (Tan and Le, 2021). Notably, EfficientNetV2 incorporates Fused-MBConv layers, which combine depthwise and pointwise convolutions for improved computational efficiency without sacrificing feature extraction. The architecture is shown in Figure 2. Additionally, squeeze-and-excitation blocks are integrated to dynamically focus on informative features within the network. Standard Conv3x3 and Conv1x1 layers are also employed for initial feature extraction and dimensionality reduction (Tan and Le, 2021).

The model takes images as input (100 × 100 x channels) and outputs a feature vector. The feature extractor is trained from scratch, with all layers set as trainable. This approach ensures that the model learns feature representations solely from the specific dataset without relying on pre-trained weights. By customizing the training process, the EfficientNetV2 architecture is tailored to the dataset, demonstrating a robust and flexible framework for feature extraction and task-specific learning.

Pooling layers play a vital role in extracting key features and reducing noise, leading to a more robust learning process. Global Average Pooling (GAP) 2D is particularly effective in reducing the dimensionality of feature maps by averaging the values within each map. This process retains critical information while minimizing the number of parameters. Dropout layers further enhance the model’s generalizability by randomly deactivating neurons during training, compelling the model to learn more robust features. A dropout rate of 0.5 indicates that half of the neurons are deactivated at random. This technique, along with learning rate adjustments, contributes to a more refined learning process for accurate hydrographic mapping. The final layer of the network is a dense layer with SoftMax activation. This fully connected layer transforms the outputs into a probability distribution for two classes (culvert and non-culvert). The SoftMax function ensures that the outputs sum to one, providing a probability interpretation. This layer signifies the final output of the network, where culvert presence is predicted. An Adam optimizer with an initial learning rate (LR) of 0.0001 was employed to update model parameters during training. The learning rate is reduced if validation accuracy stagnates, and training is halted if no improvement is observed after a predefined number of epochs. Backpropagation is utilized to calculate gradients and update weights iteratively throughout the training process. The Nebraska dataset (4,044 images) was divided into training (60%), validation (20%), and test (20%) sets for model training and evaluation. Callbacks like TensorBoard, Model Checkpoint, and Reduce LR On Plateau were employed to monitor training progress, save the best performing model, and adjust the learning rate, respectively. The training set allows the model to learn, the validation set is used to fine-tune hyperparameters, and the test set provides an unbiased measure of generalization to unseen data.

Our model development was based on Python 3.9, with TensorFlow 2.15. We integrated various Python libraries, including GDAL 3.5.0 and Rasterio 1.3.8, to support our geospatial data processing needs. Computationally, we employed Nvidia GeForce A100 40GB on google cloud for model training.

### 2.4 Model evaluation

In evaluating the EfficientNet model performance, a comprehensive set of metrics were employed, including accuracy, precision, recall, F1 score, and cross entropy loss (Equations 2–6). To effectively represent these metrics, a binary confusion matrix was utilized, distinguishing positive samples (containing flow barriers) from negative samples (without barriers). A perfect model would ideally generate only true positives and true negatives, while minimizing false positives and false negatives. True positives denote accurately identified positive predictions, false positives indicate incorrect positive predictions, true negatives represent correctly identified negative predictions, and false negatives are actual positives incorrectly marked as negative. Let TP,FP,TN, and FN denote the numbers of true positives, false positives, true negatives, and false negatives, respectively.

Accuracy is calculated by the following equation:


(2) $$\text{Accuracy}=\frac{\text{TP}+\text{TN}}{\text{TP}+\text{TN}+\text{FP}+\text{FN}}$$

which measures the overall effectiveness of the model.

Precision assesses the accuracy of the model’s positive predictions by calculating the ratio of true positives to all positive predictions, that is,


(3) $$\text{Precision}=\frac{\text{TP}}{\text{TP}+\text{FP}}$$

Recall evaluates the model’s capability to correctly identify actual positives, determined by the ratio of true positives to the total of true positives and false negatives, i.e.,


(4) $$\text{Recall}=\frac{\text{TP}}{\text{TP}+\text{FN}}$$

The F1 Score, which is the harmonic mean of precision and recall, offers a balance between these two metrics, calculated by


(5) $$F1\;\text{Score}=2\times \frac{\text{Precision}\times \text{Recall}}{\text{Precision}+\text{Recall}}$$

Cross entropy loss is a key metric in classification tasks, measuring the performance of a model whose output is a probability between 0 and 1. It quantifies the difference between two probability distributions: the actual labels and the predictions made by the model. A lower cross entropy loss indicates better model performance; that is, predictions are closely aligned with the actual labels. The formula for cross entropy loss is:


(6) $$\text{Loss}=-\sum_{i=1}^{n}y_{i}\log(p_{I}),$$

where n is the number of categories, $y_{I}$ is the truth label, and $p_{I}$ is the SoftMax probability for the ith class.

To assess the efficacy of our method, we used a recently published CNN model for classifying images containing culverts (Wu et al., 2023) as our baseline. That study employed a CNN model, which consists of four convolutional layers, one flattening layer, and two fully connected layers. The model also incorporates batch normalization between the convolution operations and the average pooling layers. The dataset’s train-test split ratio is consistent with this study.

### 2.5 CNN interpretability through XAI

Gradient-weighted Class Activation Mapping (Grad-CAM), introduced by Selvaraju et al. (2020), builds upon the original Class Activation Mapping (CAM; Zhou et al., 2016) by leveraging gradients from the final convolutional layer to generate saliency maps. These maps highlight important regions for class predictions and do not require the addition of a Global Average Pooling (GAP) layer or model retraining. In CAM the class prediction score $S_{c}$ for a given class c is determined by the weighted sum of the GAP outputs, as shown in Equation 7:


(7) $$S_{c}=\sum_{k}w^{\text{ck}}g^{k}$$

where $w^{\text{ck}}$ indicates the weight for the k-th feature map related to class c and $g^{k}$ represents the GAP output for the k-th feature map. Grad-CAM generalizes this approach by using the gradients of the class prediction score $S_{c}$ with respect to the feature maps from the last convolutional layer. The feature map values are denoted as $A_{i,j}^{k}$ at location (i, j) in the k-th feature map. The weight $w^{\text{ck}}$ is derived from the gradients of $S_{c}$ with respect to the feature maps $A^{k}$ as defined in Equation 8:


(8) $$w^{\text{ck}}=\frac{1}{Z}\sum_{i,j}\frac{\partial S_{c}}{\partial A_{i,j}^{k}}$$

where Z represents the total number of pixels in the feature map $A^{k}$. The saliency map $\text{GradCA}M_{i,j}^{c}$ is then computed by aggregating the weighted feature map values and applying a ReLU operation to capture only positive influences as shown in Equation 9:


(9) $$\text{GradCA}M_{i,j}^{c}=\text{ReLU}\;(\sum_{k}w^{\text{ck}}A_{i,j}^{k})$$

This saliency map highlights the importance of each pixel in predicting the class c based on the class prediction score $S_{c}$ and the final feature map A at each channel k. Thus, no architecture changes to the model are required. Since Grad-CAM generates a saliency map with lower resolution compared to the original input image, it can be upsampled to enhance visual clarity. Grad-CAM provides an effective XAI approach for visualizing model decisions, allowing the generation of saliency maps without the need for architectural modifications or extensive retraining of CNN models.

## 3 Results

### 3.1 Model performance

In accordance with the methods outlined, the EfficientNetV2s model was initially trained using HRDEMs for the West Fork Big Blue Watershed, Nebraska. To optimize the training, various hyperparameters were examined, such as batch sizes and values for dropouts. Among these, a batch size of 64 was found to be the most effective batch size, and 0.5 the most effective dropout value. However, it was observed that all models plateaued in performance improvements, even with adjustments in the learning rate, at approximately 75 epochs. The test accuracy of each single layer model is detailed in Table 2, suggesting that the model with TPI 21 has the highest testing accuracy (97.28%) and F1 score (97.16).

**Table 2:** Testing accuracies derived from the models with HRDEM and its derivatives.

Feature input	Accuracy	Precision	Recall	F1	Loss
HRDEM	0.9679	0.9679	0.9678	0.9679	0.1042
Curvature	0.9555	0.9556	0.9554	0.9555	0.1744
POS	0.9629	0.9699	0.9556	0.9629	0.1490
TPI 21	0.9728	0.9716	0.9716	0.9716	0.1150
TPI 3	0.9580	0.9581	0.9579	0.9581	0.1925

The total sample is 4,044 (50% False, 50% True), samples are 2,426 (60%), validate samples are 809 (20%), and test samples are 809 (20%).

Based on the single feature model performance the TPI 21 appears to be the most significant feature for accurately identifying images that contain under-road drainage crossings. This outcome highlights the effectiveness of EfficientNetV2s architecture in performing classification tasks for this problem. To quantify the individual contributions of TPI 21, HRDEM, and Positive Openness (POS) to model accuracy, a two-pronged approach is employed. First, the most influential individual features (TPI 21, HRDEM, and POS) were combined into a model for training. Second, the model utilizing all five features was trained and its performance was compared across all configurations. Table 3 demonstrates that the three-feature combination yields a superior model compared to the single best feature (Table 2). However, incorporating all five features did not lead to further improvement, conversely resulting in a decrease in model performance. Being corroborated by the results in Table 3, these findings suggest that a model utilizing only the three most impactful features (TPI 21, HRDEM, and POS) can effectively classify drainage crossing locations. Notably, the single most important feature (TPI 21) also demonstrates a high degree of accuracy in identifying these drainage crossing locations. This implies that TPI 21, HRDEM, and POS contribute comparably to the classification of drainage crossing images.

**Table 3:** Result of testing accuracy using three HRDEM-derived features and all HRDEM derived features.

Feature input	Accuracy	Precision	Recall	F1	Loss
3 Features	0.9790	0.9791	0.9790	0.9791	0.0844
All Features	0.8974	0.8974	0.8972	0.8973	0.3973

The total sample is 4,044 (50% False, 50% True), samples are 2,426 (60%), validate samples are 809 (20%), and test samples are 809 (20%).

### 3.2 Model explainability

The HRDEM model was employed to investigate how EfficientNetV2 identifies and distinguishes various topographic signatures during image analysis. To understand the model’s decision-making processes, the Grad-CAM technique (Selvaraju et al., 2020) was applied to generate saliency maps that visualize the model’s attention patterns. Grad-CAM was applied to test examples from each identified class to assess the model’s ability to recognize drainage crossings and differentiate topographic features.

**Figure 3:**
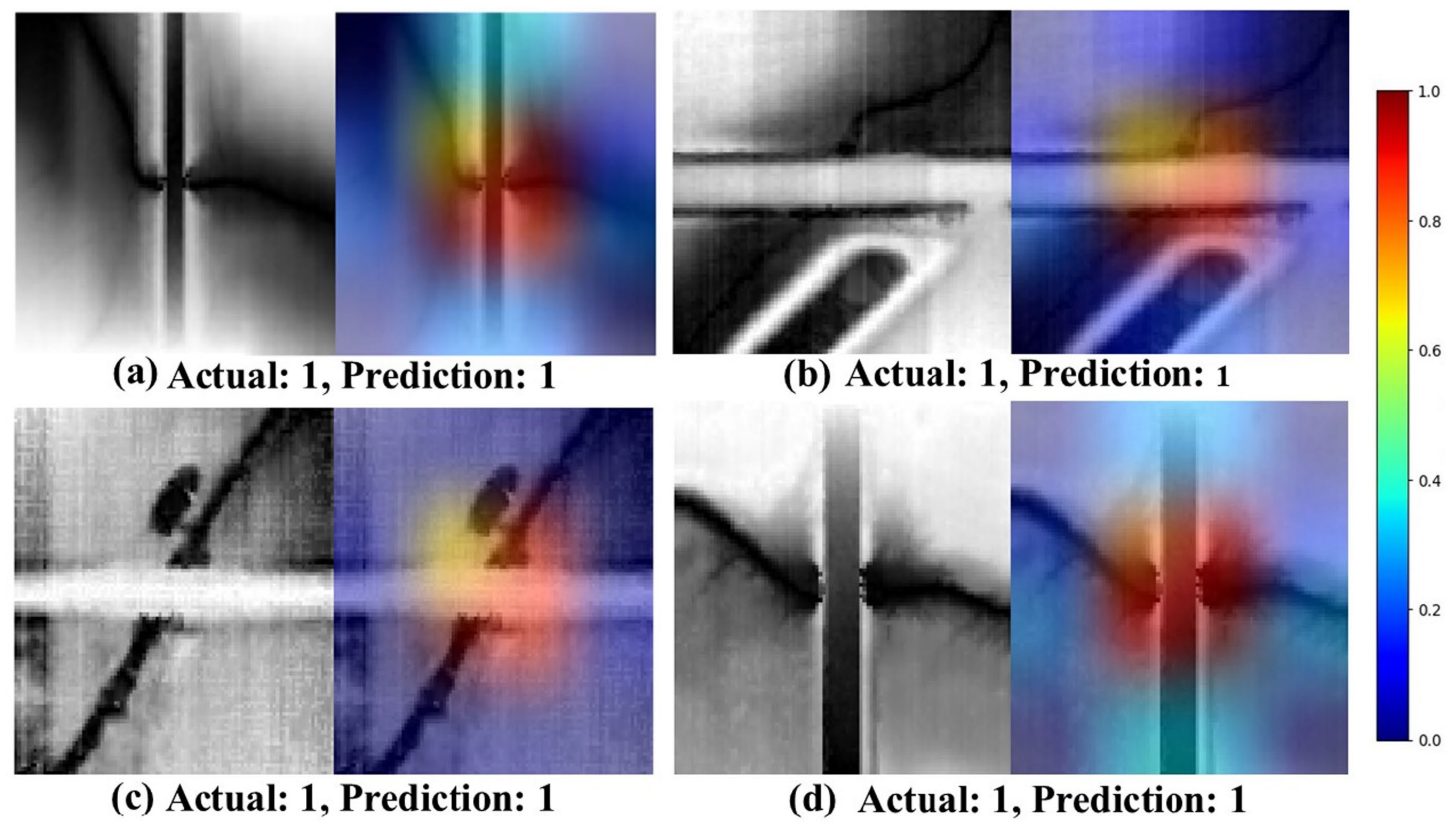
Saliency maps generated by the EfficientNetV2s model using Grad-CAM, showcasing the model’s attention patterns for correctly identified drainage crossings. In these examples **(a–d)**, the true class label is set as 1, representing the drainage crossing class. The first column displays the original grayscale images, while the second column shows the saliency maps overlaid on the original images. In the saliency maps, warmer colors (e.g., red and yellow) indicate the regions of higher importance or stronger attention by the model, suggesting areas that contribute more significantly to the classification decision. Conversely, cooler colors (e.g., blue) represent the regions with lower importance. This visualization highlights the model’s ability to focus on the key features of the drainage crossing structures during prediction.

**Figure 4:**
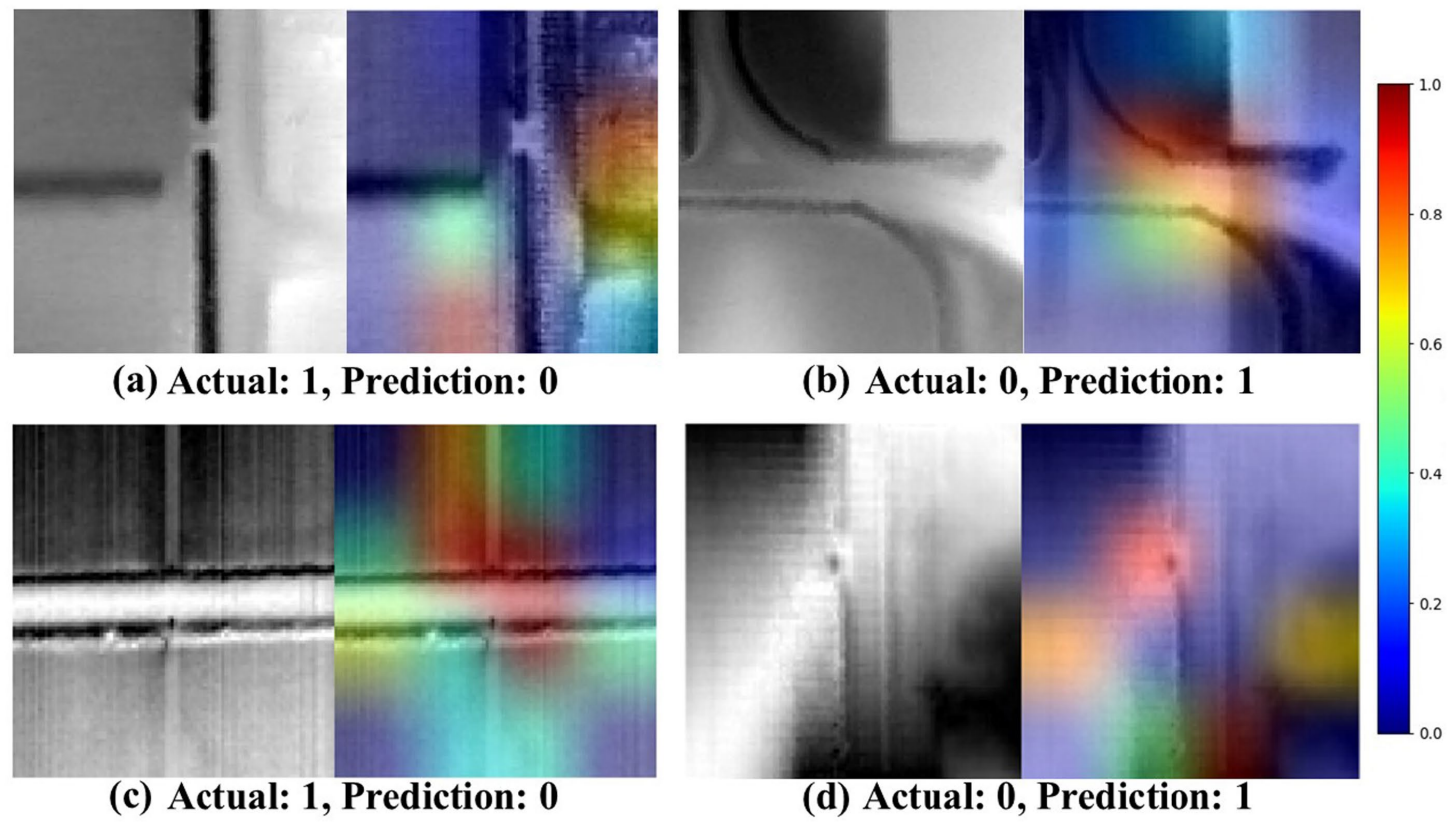
Saliency maps produced by the EfficientNetV2s model through Grad-CAM, illustrating the areas of focus when the model encounters misclassifications in drainage crossing recognition **(a–d)**. The first column displays the unaltered original image, and the second column shows a composite with the saliency map overlaid, highlighting regions where the model’s attention may have contributed to classification errors. In this visualization, true drainage crossings are labeled as class 1, while non-crossings are labeled as class 0.

Figures 3, 4 illustrate the model’s performance and decision-making process. Each figure contains four image sets, organized into two columns. The first column displays the actual (ground-truth) label, predicted label, and image identifier. The second column presents a composite image, overlaying the original image with its corresponding saliency map to highlight important regions identified by the model. In the saliency maps, regions rendered in darker shades of red and brown indicate areas with a greater influence on the model’s prediction. These visualizations provide insights into the model’s attention patterns and contribute to understanding its decision-making rationale.

In Figure 3, the highlighted regions (activations) are primarily concentrated on intersections of streams and roads, indicating the regions most relevant for identifying true culvert or drainage crossing locations. These visualizations provide valuable insights into the EfficientNetV2 model’s interpretability, highlighting the focused areas for accurately classifying drainage crossings. Figure 4 shows some instances of misclassification when the contrast between the target area and the background is minimal and unclear. In such cases, the model struggles to differentiate between drainage crossings and non-crossings, as evidenced by scattered activation patterns. The saliency maps across these figures offer insights into the model’s decision-making process, pinpointing areas that may benefit from refinement for improved classification accuracy.

### 3.3 Evaluation of model robustness

**Figure 5:**
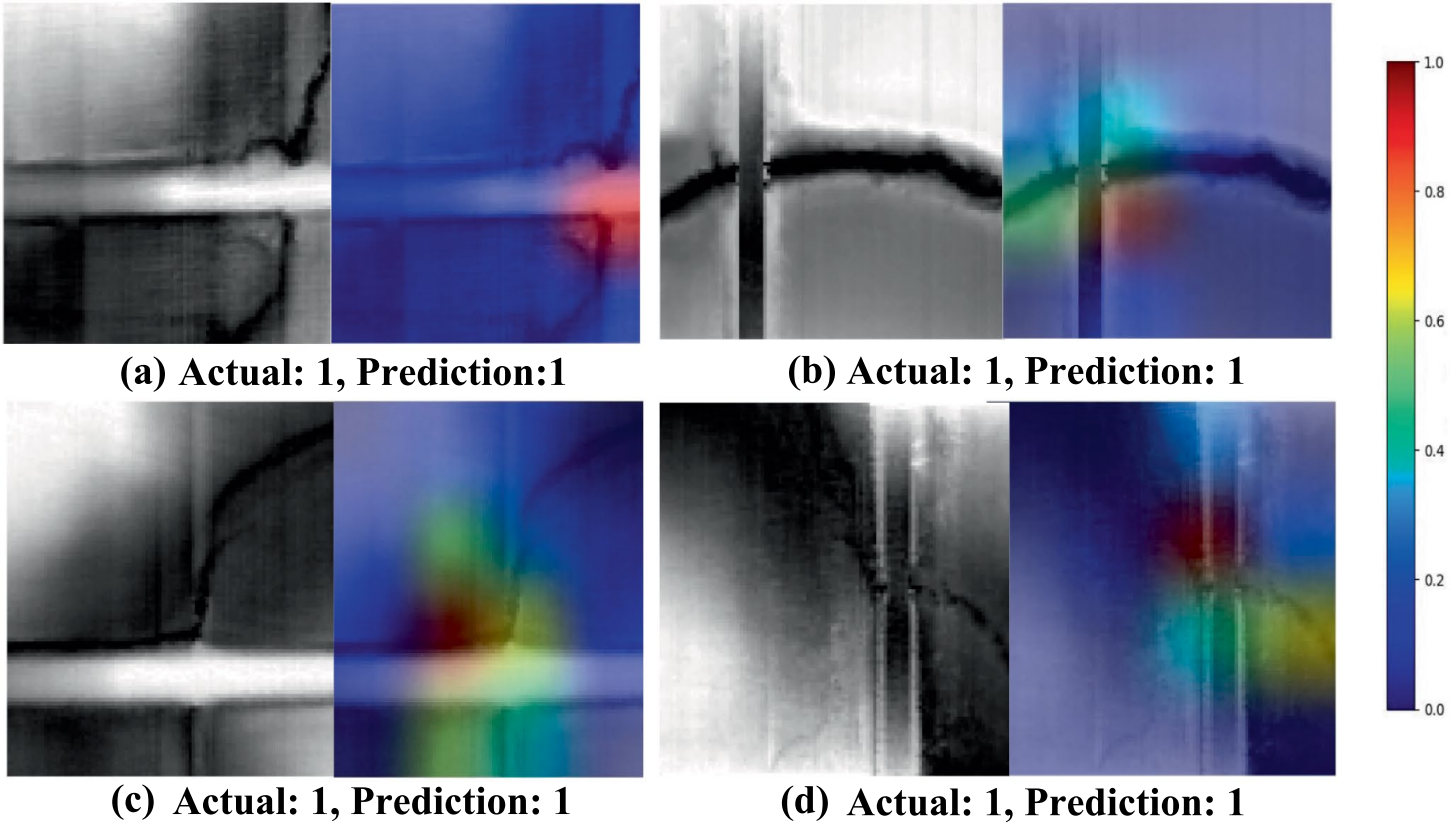
Saliency maps from the EfficientNetV2s model using Grad-CAM, showing drainage crossing detection results with varied spatial locations of targets within the images **(a–d)**. The first column in each row displays the original grayscale images, while the second column presents saliency overlays highlighting the model’s focus areas.

To further evaluate the model’s robustness, the off-center dataset was used for inferencing. This was necessary to investigate whether the model might be trained to ‘memorize’ the feature’s center location. In other words, the model should correctly detect a drainage crossing being positioned off the center of an image. The inferencing results (Figure 5) on this off-center dataset indicated that the model could detect drainage crossings at any position off the image center, demonstrating its flexibility and robustness.

## 4 Discussion

### 4.1 Multi-feature integration and model performance in drainage crossing classification

The findings from the EfficientNetV2 classification model, which leverages HRDEM-derived geomorphological features, demonstrate the effectiveness of combining multiple features for accurate deep learning-based drainage crossing detection. The model achieved the highest accuracy (97.9%) by incorporating the three most impactive input features (HRDEM, POS, and TPI21) compared to models with only single feature input. This aligns with the well-established principle of feature complementarity; that is, data from diverse sources provides a more comprehensive representation of the object of interest, ultimately leading to enhanced model performance (Xu et al., 2021; Stanislawski et al., 2021; Li et al., 2020; Barlow et al., 2022; Garcia et al., 2020).

In the DL model, each feature input contributes uniquely to drainage crossing identification. HRDEMs provide detailed elevation information, crucial for identifying potential drainage crossing locations (Bhadra et al., 2021; Talafha et al., 2021; Wu et al., 2023). POS emphasizes ridges and crests, aiding in delineating drainage pathways (Doneus, 2013). Meanwhile, TPI21 reflects broader topographic variations important for representing drainage patterns (Deumlich et al., 2010). By integrating these complementary features, the model is capable of characterizing nuanced terrain details at the location of drainage crossings.

Notably, the EfficientNetV2 models trained on individual input features also demonstrate classification accuracies comparable to the best-fit model that utilizes three feature inputs. For instance, models trained solely on TPI21, HRDEM, POS, and Curvature achieved accuracies at 0.9703, 0.9679, 0.9629, and 0.9555, respectively. These results indicate that each feature has an outstanding capability of classifying drainage crossing images, albeit with slight variations in accuracy. Among these feature inputs, TPI21 proves particularly effective due to its representation of elevation relative to neighboring cells which captures terrain features in a larger context a, emphasizing distinctive topographic patterns such as narrow valleys, lateral ridges, and drainage crossings. Importantly, a TPI window size of 21 × 21 outperformed a 3 × 3 window for drainage crossing detection. Smaller windows highlight minor elevation changes, accentuating fine topographic details. In contrast, larger windows like the 21 × 21 size emphasize broader topographic patterns, such as ridges and valleys, while smoothing minor variations (De Reu et al., 2013). These larger windows are advantageous for identifying extensive landscape features, thus making them more effective for drainage crossing detection. In essence, TPI21 provides a more holistic representation of the terrain’s morphology, potentially leading to more accurate identification of drainage crossings. The additional information embedded within TPI21 appears to be particularly beneficial for the EfficientNetV2 model’s learning process, resulting in superior performance compared to models trained on other individual input features.

### 4.2 Advancements in architecture and feature integration for improved model classification

The results obtained in this study were comparable to similar findings by Wu et al. (2023). That study developed CNN models with four convolution layers, a flatten layer, and two fully connected layers for classifying drainage crossing based on HRDEMs, aerial orthophotos, and HRDEM-derived spectral indices such as NDVI and NDWI. The model trained with a single elevation input achieved approximately 93% accuracy. In contrast, this study employed an advanced CNN model, EfficientNetV2, which demonstrated significant improvements in classification accuracy through the integration of multiple HRDEM-derived geomorphological features. The best-fit model achieved accuracy of 97.9%, underscoring the advantage of incorporating relevant complementary features to refine the model’s predictive capabilities. The findings suggest that integrating HRDEM with geomorphological features such as TPI21 and POS can improve the model’s ability to identify drainage crossings more effectively than HRDEM alone, highlighting the potential of advanced CNN architecture combined with feature diversity.

Furthermore, this study highlights the superiority of advanced DL architectures, specifically EfficientNetV2, over basic CNN models for drainage crossing classification tasks. EfficientNetV2’s advanced structure helps identify and characterize complex patterns within the drainage crossing dataset. Its scalable depth, width, and resolution facilitate more intricate feature extraction, capturing subtle terrain variations that basic CNN models may overlook. This ability to learn nuanced features results in improved performance. This advantage is further amplified by incorporating multiple geomorphological features (HRDEM, TPI21, and POS) within the EfficientNetV2 model. This combination of geomorphological features enables a more comprehensive terrain representation, underscoring the importance of both model architecture and feature diversity for enhanced performance in geospatial classification tasks.

### 4.3 XAI-based insights into model performance and misclassified image samples

Our Grad-CAM visualization analysis provided valuable insights into potential confounding regions that may influence the model’s decision-making processes. Notably, in regions where there is a low contrast between streams and roads, the model tends to focus on the road edges, as shown in Figures 4c,d. Additionally, the model successfully detected drainage patterns in images with confirmed true cases, as illustrated in Figure 3. These highlight the model’s ability to accurately capture relevant patterns, even under challenging conditions.

**Figure 6:**
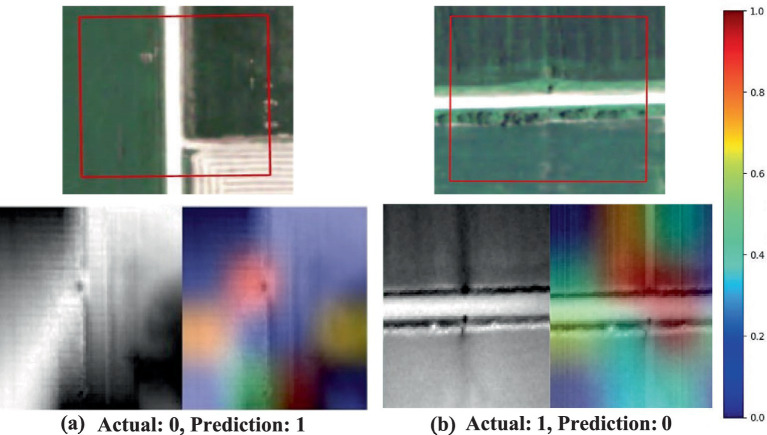
Saliency maps generated by the EfficientNetV2s model using Grad-CAM, illustrating areas of focus in cases of misclassification during drainage crossing recognition **(a,b)**. The first row shows aerial orthophotos with red boxes indicating the region of interest. In the second row, the first column shows the original grayscale image, while the second column presents a composite image with the saliency map overlaid. True drainage crossings are labeled as class 1, and non-crossings are labeled as class 0.

**Figure 7:**
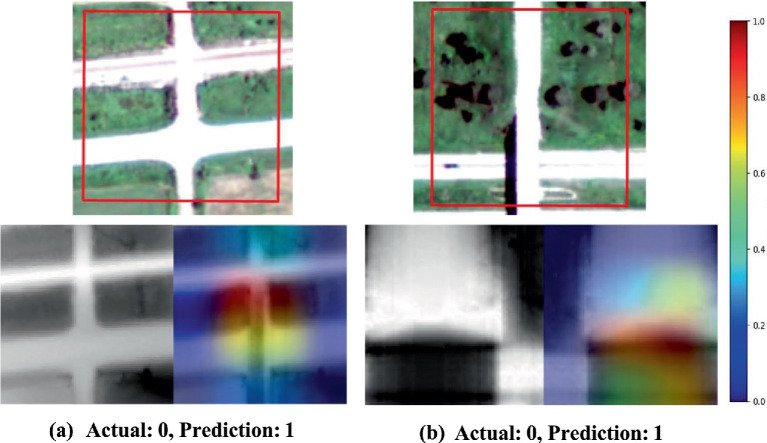
Saliency maps generated by the EfficientNetV2s model using Grad-CAM, highlighting focus areas during drainage crossing recognition **(a,b)**. The first row shows aerial orthophotos with red boxes indicating the region of interest. In the second row, the first column presents the original grayscale image, while the second column overlays the saliency map on the original. True drainage crossings are labeled as class 1, and non-crossings are labeled as class 0.

Figures 6, 7 exhibit the model’s capability of diagnosing the model’s behaviors in the cases of image misclassification. In Figure 6, the model’s activations appear concentrated along the boundaries of roads and streams. This suggests that the model may struggle to make correct classifications in regions where intersections are less distinct or where low contrast exists between the road and its surroundings. Such instances indicate potential limitations in the model’s reliance on road edge detection for accurate identification of drainage crossings. When a strong contrast exists between the road and adjacent features, the model is better suited to discern the presence of a drainage crossing. These findings highlight the critical role of feature enhancement techniques in improving classification accuracy. Techniques that could enhance contrast between roads and their surroundings, such as histogram equalization, adaptive contrast enhancement, or band-pass filtering, can substantially improve the model’s ability to accurately distinguish drainage crossings, particularly under low-contrast conditions.

Interestingly, in Figure 7, similar activation patterns are observed, where the model focuses on key intersections within the road network. However, misclassifications often arise in areas where road intersections resemble drainage crossings due to similar structural layouts. These saliency maps suggest that while the model effectively captures spatial patterns indicative of drainage crossings, it may also be confounded by complex intersections that lack the necessary hydrological characteristics, resulting in false positives. This reveals an area where the model’s sensitivity to road structures might need refinement, especially in cases where road-only intersections mimic the appearance of drainage crossings.

The misclassifications highlighted in these figures emphasize the need for incorporating additional contextual features that could aid the model in distinguishing true drainage crossings from similar-looking structures. For example, integrating geomorphological features such as positive openness (POS), and TPI could provide more clues for the model, helping it differentiate between visually similar but functionally different structures. Additionally, enhancing the model’s ability to understand texture and context beyond road edges could reduce its reliance on boundary-focused activations, leading to more accurate classifications.

The model’s consistent identification of drainage crossings across diverse spatial locations, as demonstrated by our Grad-CAM visualizations, confirms that the learned patterns are indeed predicated based on the distinctive topographic features associated with elevated roads intersecting drainage channels instead of geographic coincidence. These findings not only validate the model’s generalizability but also underscore the utility and reliability of XAI in offering comprehensive and transparent framework for accurately interpreting the model’s decision-making processes.

## 5 Conclusion

Drainage crossings are essential hydrological features with significant implications for water management, environmental planning, and infrastructure maintenance. Accurate mapping of these features is crucial for accurate hydrographic delineation and effective environmental management strategies. In this study, we applied deep learning-based image classification to identify drainage crossing locations automatically using HRDEMs combined with geomorphological features. We trained the EfficientNetV2 model, labeling images as “true” (containing a drainage crossing) or “false” (without a drainage crossing), and evaluated its performance across various feature combinations. Additionally, we applied XAI techniques to analyze the model’s attention areas, providing insights into the decision-making process for identifying drainage crossings. Key findings include:

1) The EfficientNetV2 model, utilizing HRDEM, TPI21, and POS features, achieved high accuracy (97.9%), outperforming single-feature models and highlighting the effectiveness of feature complementarity. Adding more features beyond these three led to a performance decrease, indicating an optimal balance of feature selection.
2) Grad-CAM visualizations revealed the model’s focus on key topographic features, though low-contrast areas occasionally led to misclassifications.
3) The model demonstrated robustness in identifying off-center drainage crossings, underscoring its adaptability for diverse geographic contexts.
4) Comparisons with prior studies confirm that the feature diversity and advanced CNN architectures result in improved classification accuracy.

In conclusion, this study shows the utility of integrating advanced deep learning models with carefully chosen geomorphological features for improved drainage crossing classification across various geographic contexts. Future research may be directed to refine the model further by including additional contextual features and enhancing its ability to distinguish visually similar but functionally distinct structures for improved model performance.

## Data Availability

The datasets presented in this study can be found in online repositories. The names of the repository/repositories and accession number(s) can be found in the article/supplementary material.
